# Early-Onset Molecular Derangements in the Olfactory Bulb of Tg2576 Mice: Novel Insights Into the Stress-Responsive Olfactory Kinase Dynamics in Alzheimer’s Disease

**DOI:** 10.3389/fnagi.2019.00141

**Published:** 2019-06-11

**Authors:** Mercedes Lachen-Montes, Andrea González-Morales, Maialen Palomino, Karina Ausin, Marta Gómez-Ochoa, María Victoria Zelaya, Isidro Ferrer, Alberto Pérez-Mediavilla, Joaquín Fernández-Irigoyen, Enrique Santamaría

**Affiliations:** ^1^Clinical Neuroproteomics Group, NavarraBiomed, Complejo Hospitalario de Navarra (CHN), Universidad Pública de Navarra (UPNA), Pamplona, Spain; ^2^Proteored-ISCIII Proteomics Unit, NavarraBiomed, Complejo Hospitalario de Navarra (CHN), Universidad Pública de Navarra (UPNA), Pamplona, Spain; ^3^IDISNA, Navarra Institute for Health Research, Pamplona, Spain; ^4^Department of Pathology, Complejo Hospitalario de Navarra, Pamplona, Spain; ^5^Institut de Neuropatologia, IDIBELL-Hospital Universitari de Bellvitge, Universitat de Barcelona, L’Hospitalet de Llobregat, Centro de Investigación Biomédica en Red de Enfermedades Neurodegenerativas (CIBERNED), Barcelona, Spain; ^6^Neurobiology of Alzheimer’s Disease, Department of Biochemistry, Center for Applied Medical Research (CIMA), Neurosciences Division, University of Navarra, Pamplona, Spain

**Keywords:** Alzheimer’s disease, olfactory bulb, network biology, proteomics, transcriptomics, Tg2576 mice

## Abstract

The olfactory bulb (OB) is the first processing station in the olfactory pathway. Despite smell impairment, which is considered an early event in Alzheimer’s disease (AD), little is known about the initial molecular disturbances that accompany the AD development at olfactory level. We have interrogated the time-dependent OB molecular landscape in Tg2576 AD mice prior to the appearance of neuropathological amyloid plaques (2-, and 6-month-old), using combinatorial omics analysis. The metabolic modulation induced by overproduction of human mutated amyloid precursor protein (APP) clearly differs between both time points. Besides the progressive perturbation of the APP interactome, functional network analysis unveiled an inverse regulation of downstream extracellular signal-regulated kinase (ERK1/2), and p38 mitogen-activated protein kinase (MAPK) routes in 2-month-old Tg2576 mice with respect to wild-type (WT) mice. In contrast, Akt and MAPK kinase 4 (SEK1)/ stress-activated protein kinase (SAPK) axis were parallel activated in the OB of 6-months-old-Tg2576 mice. Furthermore, a survival kinome profiling performed during the aging process (2-, 6-, and 18-month-old) revealed that olfactory APP overexpression leads to changes in the activation dynamics of protein kinase A (PKA), and SEK1/MKK4-SAPK/JNK between 6 and 18 months of age, when memory deficits appear and AD pathology is well established in transgenic mice. Interestingly, both olfactory pathways were differentially activated in a stage-dependent manner in human sporadic AD subjects with different neuropathological grading. Taken together, our data reflect the early impact of mutated APP on the OB molecular homeostasis, highlighting the progressive modulation of specific signaling pathways during the olfactory amyloidogenic pathology.

## Introduction

Together with typical symptoms, such as memory loss and behavioral disorders, Alzheimer’s disease (AD) patients present olfactory dysfunction in 90% of the cases (Attems et al., 2014; Daulatzai, 2015). However, the etiology of this smell impairment is complex and remains mostly unknown. Neuropathological studies support the hypothesis that hyperphosphorylated Tau and Aβ aggregation, present in the olfactory bulb (OB), are early and important events in the AD pathophysiology (Rey et al., 2018). It has been demonstrated that these protein deposits reflect the presence and severity of AD pathology in other brain structures (Attems et al., 2014).

Although no animal model recapitulates the entirety of human AD pathology (Sasaguri et al., 2017), some AD transgenic mouse models also present olfactory deficits. Tg2576 transgenic mice express an isoform of human amyloid precursor protein (APP) with double mutation K670N, M671L (hAPPSw; Hsiao et al., 1996). Production of Aβ40 and Aβ42 and plaques formation are observed in cortical and hippocampal areas of these mice at the age of 11–13 months (Puzzo et al., 2015). Previous reports have pointed out that the accumulation of Aβ peptide is related to age-related memory decline in these mice (Chen et al., 2000; Janus et al., 2000; Westerman et al., 2002), inducing synaptic deficits and mitochondrial imbalance (Reddy et al., 2004; Jacobsen et al., 2006). The presence of APP processing products has been characterized in the OB of 1-month-old Tg2576 mice (Lehman et al., 2003). This progressive Aβ deposition in specific olfactory structures is accompanied by behavioral deficits in odor habituation and discrimination, diminished rate of OB neurogenesis, and an altered volume of the OB granular cell layer in Tg2576 mice (Guérin et al., 2009; Young et al., 2009; Wesson et al., 2010, 2011).

Several studies using human AD brains and AD mouse models have demonstrated that the deposition of amyloid plaques is accompanied by an alteration in the OB’s molecular homeostasis (Zelaya et al., 2015; Lachén-Montes et al., 2016, 2017; Palomino-Alonso et al., 2017). However, it is still unclear how the progressive amyloidogenic pathology affects the OB functionality in the absence of plaques. In this study, we have used two high-throughput technological platforms, combining transcriptomic and proteomic approaches to ascertain the magnitude and chronology of the OB molecular remodeling in Tg2576 mice at two stages of AD: long before (2 months of age), and immediately before (6 months of age) the appearance of Aβ plaques, with respect to age-matched background strain wild-type (WT) mice. Our workflow has revealed stage-dependent molecular pathways and kinase activation dynamics that are disturbed during the initial phase of the amyloid pathology, providing basic information for understanding how olfactory molecular networks evolve as the AD pathology progresses at early stages.

## Materials and Methods

### Human Samples

According to the Spanish Law 14/2007 of Biomedical Research, informed written consent forms of Brain Bank of IDIBELL, and Neurological Tissue Bank of IDIBAPS-Hospital Clinic (Barcelona, Spain) was obtained for research purposes from relatives of patients included in this study. The study was conducted in accordance with the Declaration of Helsinki and all assessments, post-mortem evaluations, and procedures were previously approved by the Clinical Ethics Committee of Navarra Health Service. For the validation phase, 14 AD cases were distributed into different groups according to specific consensus diagnostic criteria (Braak et al., 2006; Alafuzoff et al., 2008): initial (Braak I–II), intermediate (Braak III–IV), and advanced AD stages (Braak V–VI; *n* = 4–5/group). Five cases from elderly subjects with no history or histological findings of any neurological disease were used as a control group. Eighty-five percentage of human brains considered in this study had a post-mortem interval (PMI) lower than 15 h (Table 1).

**Table 1:** General characteristics of the subjects included in this study.

Case	Sex	Age	PMI (hours)	Pathological diagnosis
**Controls**
BK-0300	F	75	20	ARP I-II
BK-1378	M	78	6	vascular encephalopathy
BK-1078	F	84	6	vascular encephalopathy, NFT I
BK-1195	F	82	8	acute ictus, cerebellar hematoma
BK-1563	M	79	15	acute ictus, AgD II
**initial AD**
A13/70	F	79	10	AD II/A
A14/29	F	78	3.5	AD II/A
A14/33	M	62	9.5	AD II/0
A14/52	M	70	3	AD II/0
**intermediate AD**
A12/42	F	82	17	AD IV/A
A12/47	M	81	5	AD III/A
A12/48	M	84	12	AD IV/A
A12/54	M	89	3	AD IV/A
A15/17	M	84	20	AD III/A
**Advanced AD**				
CS-1445	F	73	3.5	AD VI/C + moderate CAA
CS-0662	M	75	4	AD VI/C
CS-0535	F	81	4.5	AD VI/C
CS-0673	M	75	4.25	AD VI/C
CS-1232	M	84	5	AD VI C + CAA

*PMI, post-mortem interval; AD/ARP Alzheimer’s disease related pathology; NFT: neurofibrillary tangles I-VI according to Braak, amyloid plaques A–C according to CERAD; CAA: cerebral amyloid angiopathy; AgD: argyrophilic grain disease*.

### Materials

The following reagents and materials were used. Electrophoresis reagents were purchased from Bio-rad and Trypsin from Promega. Antibodies used during the study are summarized in Table 2.

**Table 2:** Antibody used in this study.

Antibodies	Product number	Distributor
anti-APP	2450	Cell signaling
anti-MEK1/2	9126
anti-phospho-MEK1/2 (S217/221)	9154
anti-ERK1/2	9102
anti-phospho-ERK1/2 (T202/y204)	4370
anti-Akt	4685
anti-phospho-Akt (S473)	4060
anti-p38 MAPK	9212
anti-phospho-p38 MAPK (T180/Y182)	9211
anti-phospho-ATF2 (T71)	5112
anti-SAPK/JNK	9252
anti-phospho-SAPK/JNK (T183/Y185)	9255
anti-SEK1	9152
anti-phospho-SEK1 (S257/T261)	9156
anti-PKA C-alpha	4782
anti-phospho-PKA C (T197)	5661
anti-PP5	2289
anti-PDK1	3062
anti-phospho-PDK1 (S241)	3061
anti-phospho-PKC pan (T514)	9379
anti-phospho-FAK (Y576/577)	3281
anti-Phb1	2426
and anti-Phb2	14085
Anti-PKC-pan	SAB4502356	Sigma Aldrich

### Animals

Transgenic mice (Tg2576) overexpressing hAPP, carrying the Swedish (K670N/M671L) familial AD mutation and under control of the prion promoter (Hsiao et al., 1996), were used. Mice were on an inbred C57BL/6/SJL genetic background. The animals were maintained in positive pressure-ventilated racks at 25 ± 1°C with a 12 h light/dark cycle, fed *ad libitum* with a standard rodent pellet diet (Global Diet 2014; Harlan Laboratories, Indianapolis, IN, USA) and had free access to filtered and UV-irradiated water. All animal care and experimental procedures were in accordance with European and Spanish regulations (86/609/CEE; RD1201/2005) and were approved by the Ethical Committee of the University of Navarra (no. 018/05). Twenty-four animals, divided into two sets, were used for proteomics and transcriptomics analysis (12 mice/approach), with at least three WT and three Tg2576 transgenic mice per stage (2- and 6-month-old). Additionally, 14- and 18-month-old animals were used for immunohistochemistry and cell survival routes signaling analysis. Table 3 summarizes the number and the purpose for each of the animals. The progressive development of AD signs in our colony has been previously described (Cuadrado-Tejedor and García-Osta, 2014). We have previously observed that behavior (Morris Water Maze test, MWM) is completely normal and amyloid levels are equal to wild type at 2 months of age. At 6 months of age, mice show impaired cognitive functions in the contextual fear conditioning test, coinciding with the increased cortical and hippocampal soluble β amyloid (Aβ) levels. At 12 months, the impairment in MWM is present in most of the mice, but few are normal and with less plaques (but they are present); and finally, in aged mice (17–18 months), the pathology is robust and 100% of mice shows plaques and MWM impairment.

**Table 3:** Number of animals used in each experimental approach.

Mice	Proteomics	Transcriptomics	Ab burden analysis	Signaling routes study
3 month-old wild type	3	3	3	3
3 month-old Tg2576	3	3	3	3
6 month-old wild type	3	3	3	3
6 month-old Tg2576	3	3	3	3
14 month-old wild type	–	–	3	–
14 month-old Tg2576	–	–	3	–
18 month-old wild type	–	–	–	3
18 month-old Tg2576	–	–	–	3

### Sample Preparation for Proteomic Analysis

Murine OB specimens were homogenized in lysis buffer containing 7 M urea, 2 M thiourea, 50 mM DTT. The homogenates were spinned down at 100,000 × g for 1 h at 15°C. Protein concentration was measured in the supernatants with the Bradford assay kit (Bio-Rad).

### Olfactory Bulb Proteomics

Sample preparation, Protein Digestion and Peptide iTRAQ Labeling were performed as previously described (Zelaya et al., 2015; Lachén-Montes et al., 2017). Briefly, each tryptic digest was labeled according to the manufacturer’s instructions with one isobaric amine-reactive tags as follows: set 1 (experiment with 2-months-old mice): Tag113, WT-1; Tag114, WT-2; Tag115, WT-3; Tag116, Tg2576–1; Tag117, Tg2576–2; Tag118, Tg2576–3, and Set 2 (experiment with 6-months-old mice): Tag113, WT-1; Tag114, WT-2; Tag115, WT-3; Tag116, Tg2576–1; Tag117, Tg2576–2; Tag118, Tg2576–3. After 2 h of incubation, the set of labeled samples were pooled and evaporated in a vacuum centrifuge. To increase the proteome coverage, the peptide pool was submitted to cation exchange chromatography using spin Columns (Pierce). Twelve fractions were collected (from 5 mM to 250 mM KCl), concentrated using C18 zip tip solid phase extraction (Millipore), evaporated under vacuum and reconstituted into 10 μl of 2% acetonitrile, 0.1% formic acid, 98% MilliQ-H20 prior to mass spectrometric analysis. Peptide mixtures were separated by reverse phase chromatography and analyzed by mass-spectrometry as previously described (Palomino-Alonso et al., 2017). The raw MS/MS spectra search were processed using the MaxQuant software (v.1.5.8.3; Tyanova et al., 2016a) and searched against the Uniprot proteome reference for Mus musculus (Proteome ID: UP000000589, May 2017). The parameters used were as follows: initial maximum precursor (25 ppm), fragment mass deviations (40 ppm); variable modification (methionine oxidation and N-terminal acetylation) and fixed modification (MMTS); enzyme (trypsin) with a maximum of one missed cleavages; minimum peptide length (seven amino acids); false discovery rate (FDR) for PSM and protein identification (1%). Frequently observed laboratory contaminants were removed. Protein identification was considered valid with at least one unique or “razor” peptide. The protein quantification was calculated using at least two razor + unique peptides, and statistical significance was calculated by a two-way Student *t*-test (*p* < 0.05). A 1.3-fold change cut-off was used. Proteins with iTRAQ ratios below the low range (0.77) were considered to be down-regulated, whereas those above the high range (1.3) were considered to be upregulated. The Perseus software (version 1.5.6.0; Tyanova et al., 2016b) was used for statistical analysis and data visualization. Search results files and MS raw data were deposited to the ProteomeXchange Consortium^1^ *via* the PRIDE partner repository (Vizcaíno et al., 2014) with the dataset identifiers PXD007813 (username: reviewer31643@ebi.ac.uk; password: gMcgfWzD).

### Olfactory Bulb Transcriptomics

OB Transcriptomics-Maxwell^®^ 16 simplyRNA Kit (Promega) was used to extract the OB mRNAs from Tg2576 mice and WT littermates. The sense cDNA was fragmented and biotinylated using the Affymetrix Clarion S Pico assay (902932). Affymetrix mouse Clarion S chips were used according to the manufacturer protocols. Hybridization, washing, staining, scanning, and data analysis (Irizarry et al., 2003) were performed as previously described (Lachen-Montes et al., 2017). As in other transcriptomic studies performed in AD brains (Silva et al., 2012; Cuadrado-Tejedor et al., 2016), we worked with a *p*-value < 0.01 (without using any method for multiple testing correction). Microarray data files were submitted to the GEO (Gene Expression Omnibus) database and are available under accession number GSE106643.

### Bioinformatics

The identification of specifically dysregulated regulatory/metabolic networks in Tg2576, OBs was analyzed using QIAGEN’s Ingenuity^®^ Pathway Analysis (IPA; QIAGEN Redwood City^2^). The software generates significance values (*p*-values) between each biological or molecular event and the imported molecules based on the Fisher’s exact test (*p* ≤ 0.05). The IPA comparison analysis considers the signaling pathway rank according to the calculated *p*-value and reports it hierarchically.

### Immunoblotting Analysis

Equal amounts of OB protein (5 μg) were resolved in 4%–15% TGX stain-Free gels (Bio-Rad). OB proteins derived from murine and human samples were electrophoretically transferred onto nitrocellulose membranes using a Trans-blot Turbo transfer system (up to 25V, 7 min; Bio-Rad). Equal loading of the gels was assessed by stain free digitalization and by Ponceau staining. Western-blotting was performed as previously described (Lachén-Montes et al., 2017). After densitometric analyses (Image Lab Software Version 5.2; Bio-Rad), optical density values were expressed as arbitrary units and normalized to total stain in each gel lane.

### Immunohistochemistry

Under xylazine/ketamine anesthesia, animals were perfused transcardially with saline for 3 min at a 11 ml/min flow, and 4% paraformaldehyde in phosphate buffered saline (PBS) for 2 min at a 9 ml/min flow. After perfusion, brains were removed, post-fixed in 4% paraformaldehyde for 1 h at room temperature and cryoprotected in 30% sucrose solution in PBS overnight at 4°C. Brains were sliced into 40-μm-thick coronal sections along the rostral axis with a freezing microtome (Leica, Germany) and collected in 0.125 M PBS containing 2% dimethylsulphoxide (Sigma), 20% glycerin (Panreac) and 0.05% sodium azide, and stored at −20°C until their subsequent analysis. Five free-floating tissue sections, comprising the OB of four animals per age group (2-, 6- and 14-month old), were processed for immunohistochemistry. The sections were washed (3 × 10 min) with a solution buffer containing PBS 0.125 M (pH 7.4), 0.5% Triton X-100 and 0.1% BSA. After washing, sections were treated with methanol and H_2_O_2_ to inhibit endogenous peroxidase activity and incubated in 70% formic acid for 5 min to expose the epitope. Subsequently, the sections were incubated overnight with a primary mouse antibody (6E10) raised against human Aβ (amino acids 1–16; BioLegend, San Diego, CA, USA) diluted 1:1,000 in PBS 0.125 M (pH 7.4), 0.5% Triton X-100, 0.1% BSA and 5% normal goat sera. After washing (3 × 5 min) in PBS, sections were incubated for 30 min with biotinylated goat anti-mouse secondary antibody (DakoCytomation, Glostrup, Denmark) diluted 1:500 in PBS. The sections were then processed using the avidin–biotin-peroxidase complex (Vectastain kit, Vector Laboratories, Burlingame, CA, USA) and reacted with 0.05% 3,3′-diaminobenzidine tetrahydrochloride (DAB) and 0.015% H_2_O_2_ in 50 mM Tris HCl, pH 7.2. After washing in deionized water, sections were mounted on gelatinized slides, counterstained with Thionine at 60°C (Panreac Quimica, Barcelona, Spain) and cover-slipped with DPX (VWR, Dublin, Ireland). With respect to human OB samples, formalin-fixed, paraffin-embedded tissue sections from OB (derived from controls and AD cases) were sectioned at 5 μm and counterstained with hemeatoxylin for immnuhistochemistry analysis with anti-SEK1 (ref. 9152; 1:50), anti-phospho-SEK1 (S257/T261; Ref. 9156; 1:250), anti-protein kinase A (PKA) C-alpha (ref. 4782; 1:50), anti-phospho-PKA C (T197; ref. 5661; 1:250). Visualization was performed by an automated slide immunostainer (Leica Bond Max) with BondPolymer Refine Detection (Leica Biosystems Newcastle Ltd., Newcastle upon Tyne, UK).

## Results

### APP Overproduction Induces Early, and Time-Dependent Molecular Derangements in the Olfactory Bulb of Tg2576 Mice

**Figure 1:**
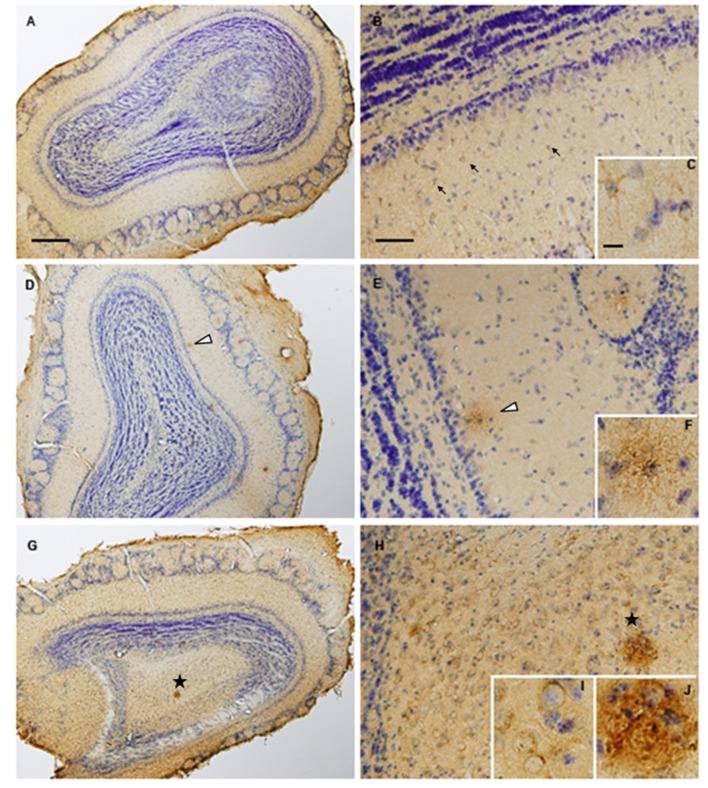
Olfactory β-Amyloid Aβpathology increases with the disease progression in TG2576 mice. Olfactory bulbs (OBs) were harvested from 2-**(A–C)**, 6-**(D–F)** and 14-**(G–J)** month-old Tg2576 mice. Intraneuronal Aβ immunoreactivity can be observed in 2-month-old mice (arrow heads; panel **B** and more detailed in panel **C**). OB samples from 6-month-old animals (panels **D–F**) shows moderate Aβ deposition in form of diffuse plaques (asterisk). By contrast, mature plaques (asterisk in panels **G**,**H** and insert **J**) and vascular Aβ (insert **I**) is evident in 14-month-old Tg2576. Scale bars (500 μm for panels **A,D,G**, 100 μm for panels **B,E,H**) or 10 μm **(C,F,I,J)**.

**Figure 2:**
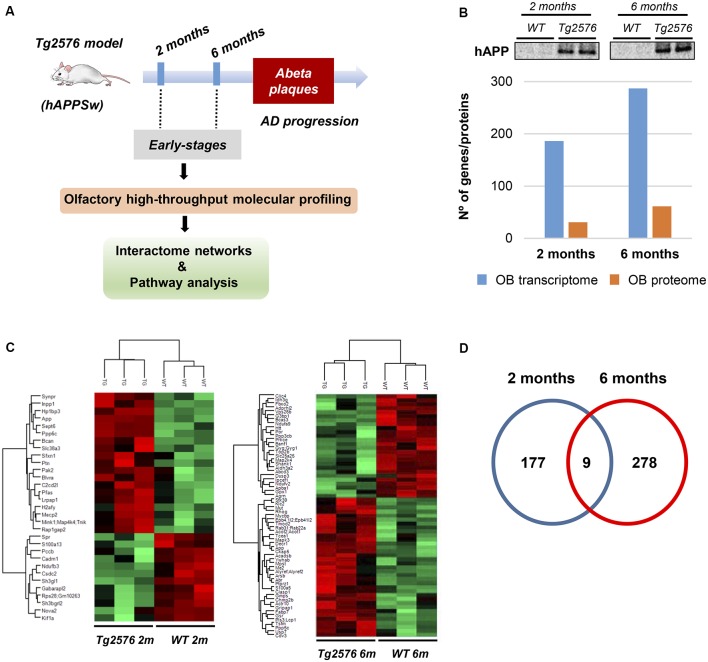
Dual-omic approach to characterize the OB molecular homeostasis between 2-month-old and 6-month-old Tg2576 mice. **(A)** An overview of the experimental workflow used in this study. **(B)** Differential molecular profiling detected by-omics in Tg2576 OBs (2 and 6 months of age). The OB protein expression levels of amyloid precursor protein (APP) at early Alzheimer’s disease (AD) stages in Tg2576 mice is shown. **(C)** Heat maps representing the degree of change for the differentially expressed proteins (Supplementary Table S2) between 2-and 6-month-old Tg2576 mice respect to wild-type (WT) littermates. Red and green, up- and down-regulated proteins, respectively. **(D)** Venn diagram for the differentially expressed genes detected in Tg2576 mice at both time points (Supplementary Table S1).

First, we analyzed the olfactory Aβ pathology in TG2576 mice. As shown in Figure 1, intraneuronal Aβ immunoreactivity was observed in 2-month-old transgenic mice, detecting Aβ deposition in the form of diffuse plaques and mature plaques at the age of 6 and 14 months, respectively (see Supplementary Figure S1 for more details). As the primary aim of our study was to analyze early-onset molecular derangements in the OB of Tg2576 mice, we deeply monitored OB molecular disturbances at two time-points (2 and 6 months), using high-throughput molecular technologies (Figure 2A). At both time-points, Tg2576 mice displayed abundant full-length human APP expression in the OB (Figure 2B). To examine the consequences of initial incremental accumulation of APP on OB molecular homeostasis, we applied proteomics and transcriptomics with the final goal to decipher novel information about the OB site-specific molecular signature at early AD stages in 2-month and 6-month-old Tg2576 mice. To analyze the potential differences in olfactory molecular expression profiles, OB specimens for each experimental group (Tg2576 and WT mice) were subjected into chemical tags (iTRAQ) coupled to tandem mass spectrometry and into the RNA microarray platform. With respect to transcriptome-wide analysis, 187 protein-coding genes were differentially regulated in the OB of 2-month-old Tg2576 mice (46 down- and 141 up-regulated genes with respect to WT mice), whereas 287 differentially expressed genes were found in 6-month-old Tg2576 OBs (107 down- and 180 up-regulated genes with respect to WT mice; Figure 2B, and Supplementary Table S1). In the proteomic phase, 1,605 and 1,752 proteins were quantified at 2 and 6 months, respectively. The expression levels of 31 proteins were found to be significantly different between 2-month-old WT and Tg2576 mice (12 down- and 19 up-regulated proteins with respect to WT mice), and 61 differentially expressed proteins were detected at 6 months of age (26 down- and 35 up-regulated proteins with respect to WT animals; Figures 2B,C, and Supplementary Table S2). To partially validate our quantitative LC-MS/MS approach, the increment in Serine/threonine-protein phosphatase 5 (PP5), a phosphatase that protects neurons against Aβ toxicity (Sanchez-Ortiz et al., 2009), was verified by Western-blotting in 6-month-old Tg2576 OB (Supplementary Figure S2A). Interestingly, the genes and proteins affected between both stages varied widely, with only one protein (APP) and nine genes common to the two time-points (Figure 2D). Of these nine genes, four were co-downregulated (FOS, ARC, NPAS4, RGSL1) and five were co-upregulated (STMN4, STMN2, F3, HIF3a, EMB).

### Functional Modules Progressively Disrupted by Human Mutated APP in the Olfactory Bulb of Tg2576 Mice

**Figure 3:**
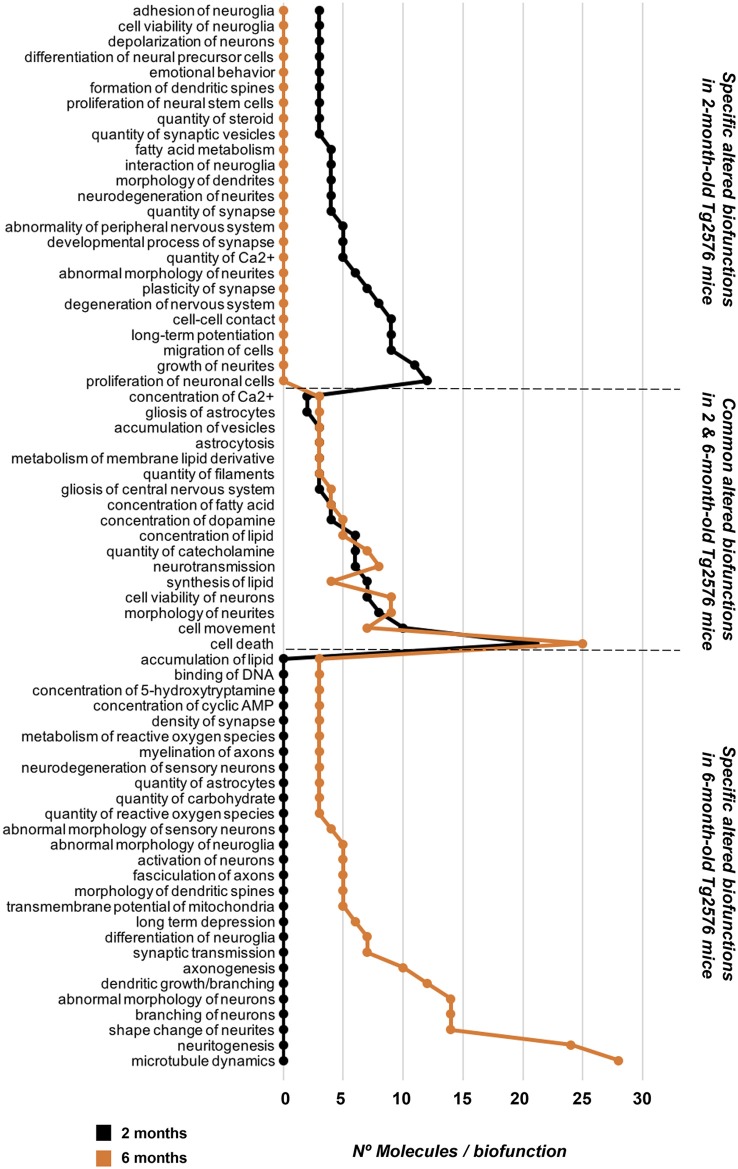
Profiling of molecular biofunctions potentially altered in the OB of Tg2576 mice. Functional analysis was performed with IPA software using exclusively the database information of experimental and predictive origin regarding central nervous system to be confident about the potential affected signaling pathways (see Supplementary Table S3 for details).

**Figure 4:**
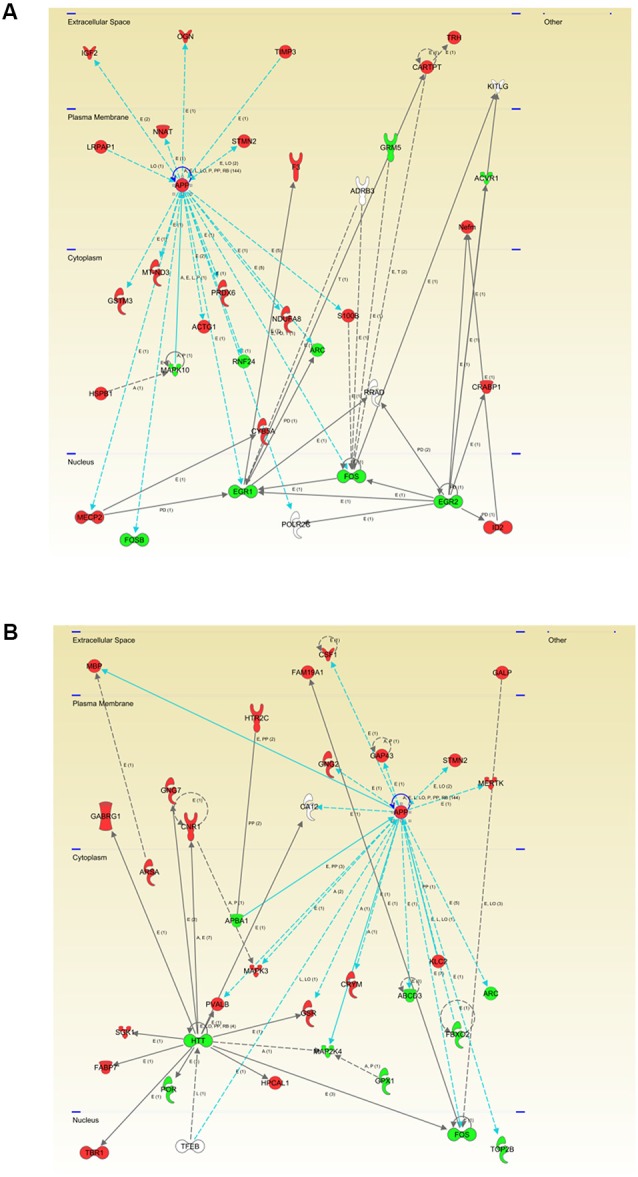
Modulation of the APP functional interactome in Tg2576 mice at the level of OB. Adaptation of APP functional network in Tg2576 OBs at 2 months of age **(A)** and 6 months of age **(B)**. Relationships between differential expressed genes/proteins and APP functional interactors are represented with blue lines. Continuous lines represent direct interactions, while discontinuous lines correspond to indirect functional interactions. Up-regulated molecules in red, and down-regulated molecules in green (See complete legend at: http://qiagen.force.com/KnowledgeBase/KnowledgeNavigatorPage#).

To characterize in detail the proteogenomic modulation induced by the presence of human mutated APP at pre-plaque stages in the OB, differential proteomic and transcriptomic datasets were merged, and functionally analyzed across specific biological functions (see Supplementary Table S3). Functional bioinformatic analysis revealed that the accumulation of APP resulted in disturbances of statistically over-represented molecular processes directly relevant to adhesion (*p*-val: 0.0001), viability (*p*-val: 0.01), and interaction of neuroglia (*p*-val: 0.0001), quantity (*p*-val: 0.003), development (*p*-val: 0.04), and plasticity of synapse (*p*-val: 0.001), long-term potentiation (*p*-val: 0.006), growth of neurites (*p*-val: 0.03), and neuronal proliferation (*p*-val: 0.04) in 2-month-old Tg2576 mice (Figure 3). In 6-month-old Tg2576 mice, the olfactory amyloid pathology predominantly resulted in the significant alteration of microtubule dynamics (*p*-val: 0.0001), neuritogenesis (*p*-val: 0.0005), axonogenesis (*p*-val: 0.007), synaptic transmission (*p*-val: 0.01), transmembrane potential of mitochondria (*p*-val: 0.00001), metabolism of ROS (*p*-val: 0.02), and neurodegeneration of sensory neurons (*p*-val: 0.02) between others (Figure 3). However, functional commonalities focused on synthesis, and concentration of lipids/fatty acids, cell death, astrocytosis, and accumulation of vesicles were also detected in 2- and 6-month-old Tg2576 OBs (Figure 3, and Supplementary Table S3). Considering that the discovery of unexpected connections between seemingly unrelated molecules and human mutated APP is a straightforward approach for the identification of novel AD causative targets involved in early olfactory neurodegeneration, we explored whether highly expressed APP isoform was potentially interconnected with differential molecular mediators identified in our proteogenomic approach. For that, functional interactomes were generated using IPA software. Interestingly, 35 differential functional interactors for APP were identified in the OB of Tg2576 mice at pre-plaque stages, suggesting the involvement in related biological functions (Figure 4). Specifically, at 2 months of age, olfactory APP is central to an interconnected molecular network composed of targets with specific subcellular distribution: (i) IGF2, OGN, TIMP3 at extracellular level; (ii) LRPAP1, NNAT, STMN2 in the plasma membrane; (iii) cytoplasmic GSTM3, MT-ND3, MAPK10, ACTG1, PRDX6, RNF24, NDUFA8, ARC, and S100B; and (iv) MECP2, FOSB, EGR1, FOS, EGR2 in the nucleus (Figure 4A). At 6 months of age, the olfactory APP interactome completely varied at extracellular (MBP, and CSF1), plasma membrane (GNG2, GAP43, STMN2, MERTK), cytoplasmic (APBA1, PVALB, MAPK3, GSR, CRYM, MAP2K4, ABCD3, KLC2, FBXO2, ARC), and nuclear (FOS, TOP2B) levels (Figure 4B).

**Figure 5:**
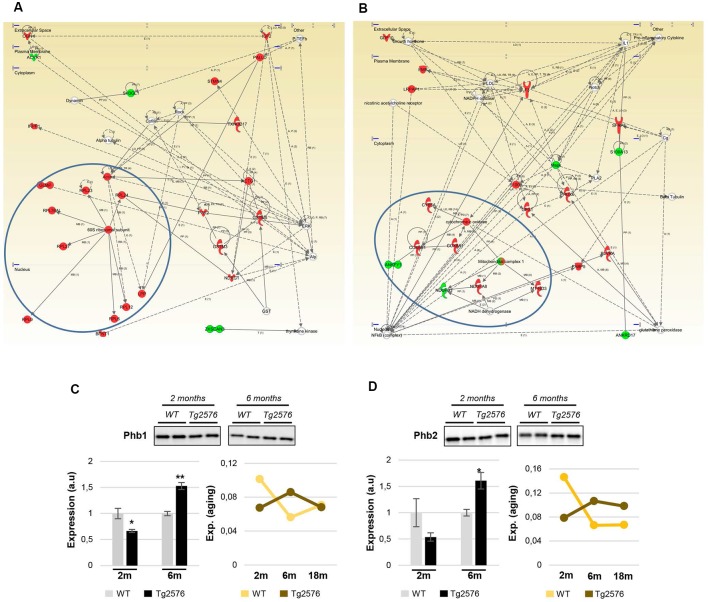
Protein synthesis, and mitochondrial homeostasis are early compromised in Tg2576 at olfactory level. **(A)** Molecular network representing the up-regulation of ribosomal proteins (blue circles) in the OB of 2-month-old Tg2576. **(B)** Molecular network highlighting the dysregulation of specific components of the mitochondrial respiratory chain (Complex I, and Complex VI subunits) in the OB of 2-month-old Tg2576. **(C)** Time-dependent disruption of the olfactory Phb1 in Tg2576 mice. Phb expression was monitored by Western-blotting. **(D)** Time-dependent disruption of the olfactory Phb2 in Tg2576 mice. Phb expression was monitored by Western-blotting. Equal loading of the gels was assessed by stain-free digitalization. Panels show histograms of band densities. Data are presented as mean ± standard error of the mean (SEM) from three independent OB samples per group (*P < 0.05 vs. control group; **P < 0.01 vs. control group). Right graphs represent the expression of both Phb subunits during the aging process in WT and Tg2576 mice (2-, 6-, and 18-month-old).

Additional integrative networks unveiled an early disruption of EIF2 signaling based on the up-regulation of a subset of ribosomal proteins (Figure 5A), suggesting that olfactory protein synthesis is compromised in 2-month-old Tg2576 mice. Furthermore, a dysregulation of specific subunits, corresponding to the mitochondrial complexes I and VI, was evidenced in the OB of 2-month-old Tg2576 mice, suggesting an olfactory mitochondrial impairment (Figure 5B). It is well-known that prohibitin complex (constituted by Phb1 and Phb2) is a mitochondrial inner membrane-bound chaperone that participates in the mitochondrial respiratory complex assembly, modulates mitochondrial dynamics, and exerts beneficial effects on neurons by reducing free radical production (Artal-Sanz and Tavernarakis, 2009; Zhou et al., 2012). Subsequent experiments were performed to monitor the expression of both Phb subunits in the OB from Tg2576 mice. As shown in Figure 5C, a significant drop in Phb1 levels was evidenced in 2-month-old Tg2576 OBs, increasing its levels at 6 months of age with respect to WT littermates. Interestingly, a similar trend was also observed for the Phb2 protein in Tg2576 mice (Figure 5D). OB Phb complex was also independently evaluated in WT and Tg2576 mice during aging. With respect to data obtained at 2 months of age, Phb1 and Phb2 protein levels are decreased at 6 and 18 months in WT animals, maintaining constant levels in Tg2576 mice during the aging process (Figures 5C,D). These data indicate that Phb complex is an early target of human mutated APP, suggesting that the stable maintenance of Phb levels may help to counteract the oxidative stress present in olfactory neurons during AD progression in Tg2576 mice.

### Human Mutated APP Modifies the Olfactory Signaling Routes in a Stage-Dependent Manner

**Figure 6:**
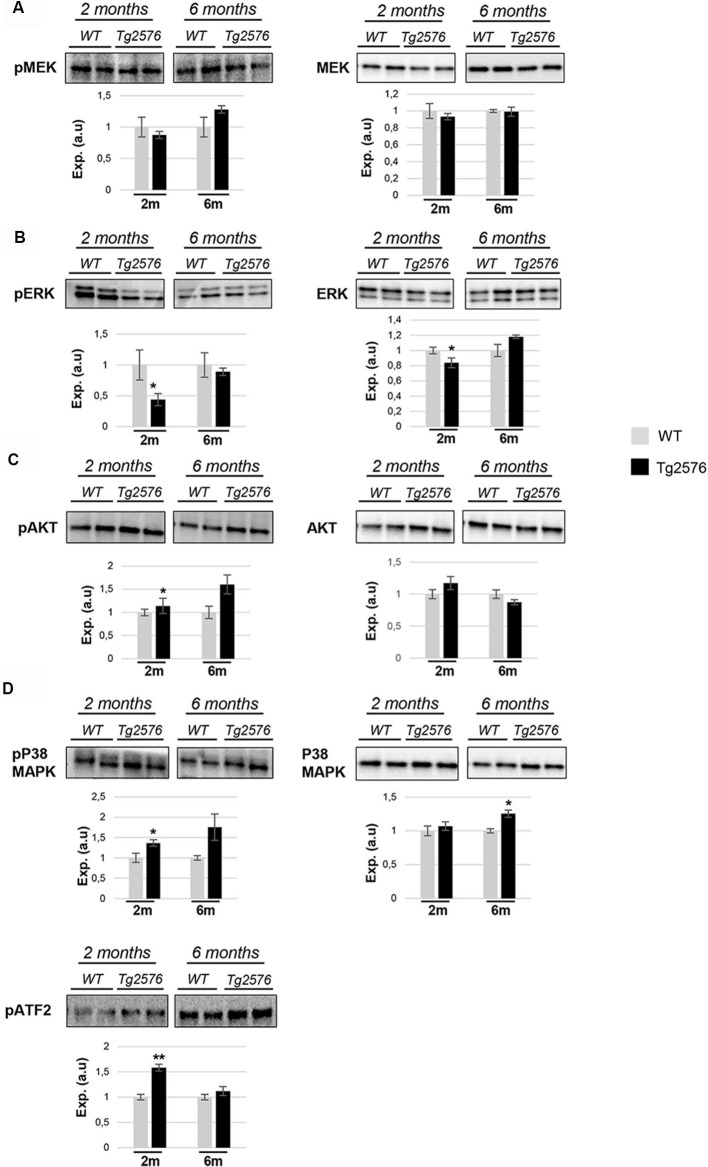
APP overproduction affects the activation state of olfactory ERK1/2, Akt, and p38 mitogen-activated protein kinase (MAPK) at early AD stages in Tg2576 mice. Levels and residue-specific phosphorylation of MEK1/2 **(A)**, ERK1/2 **(B)**, Akt **(C)**, and p38 MAPK-ATF2 axis **(D)**. Equal loading of the gels was assessed by stain-free digitalization. Panels show histograms of band densities. Data are presented as mean ± SEM from three independent OB samples per group. *P < 0.05 vs. control group; **P < 0.01 vs. control group.

**Figure 7:**
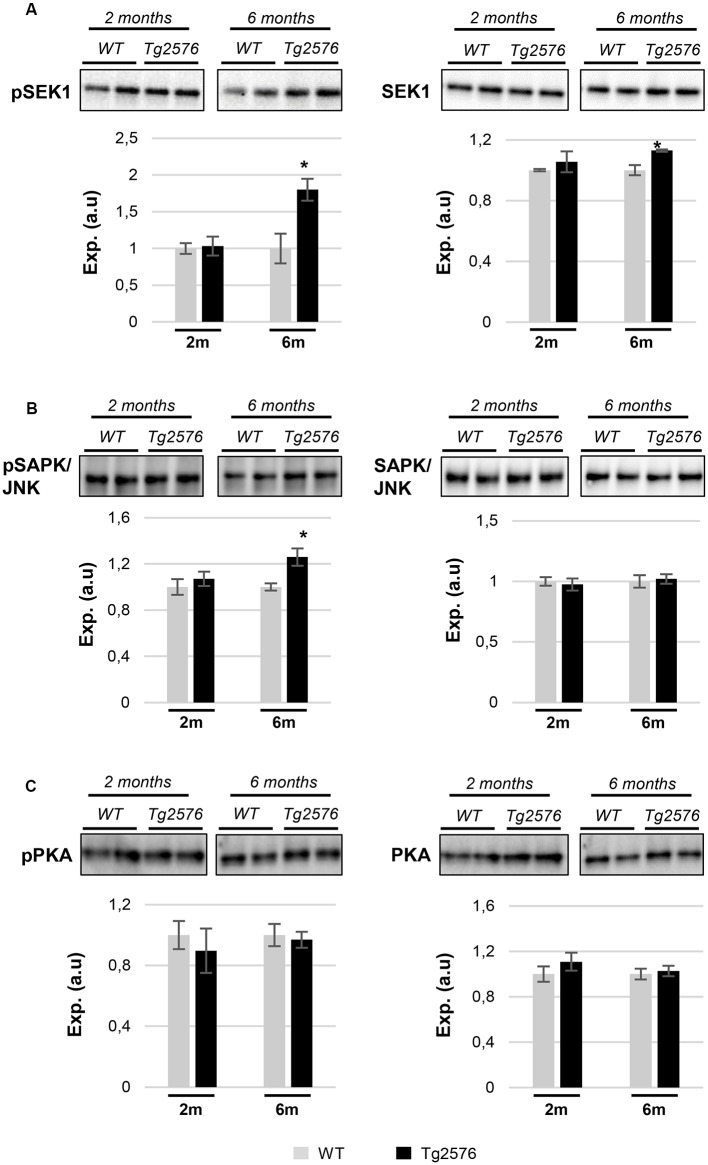
APP overproduction specifically modulates the activation of the SEK1/MKK4-stress-activated protein kinase (SAPK)/JNK axis in 6-month-old Tg2576 mice. Time-dependent expression of total and phosphorylated levels of SEK1 **(A)**, SAPK/JNK **(B)**, and protein kinase A (PKA; **C**). Equal loading of the gels was assessed by stain-free digitalization. Panels show histograms of band densities. Data are presented as mean ± SEM from three independent OB samples per group. *P < 0.05 vs. control group.

The molecular network analysis also pointed out functional links between APP and a cluster of survival kinases, such as ERK, p38 mitogen-activated protein kinase (MAPK), and Akt (Supplementary Figure S3). Subsequent experiments were performed to analyze the activation state of MAPKs, Akt, and p38 MAPK at pre-plaque stages in the OB of Tg2576 mice. A downstream inactivation in the MAPK pathway at the level of ERK was specifically observed in 2-month-old Tg2576 mice (Figure 6B), being the activation state of upstream MEK unaffected (Figure 6A). Moreover, olfactory Akt was specifically activated in 6-month-old transgenic mice (Figure 6C). In addition, Western-blot analysis revealed an increase in the activation status of OB p38 MAPK in 2-month-old transgenic mice (Figure 6D). This early activation was accompanied by a paralleled increment in the phosphorylation status of ATF2 (Figure 6D), a well-known downstream substrate of p38 MAPK (Puig et al., 2004). To complement our signaling mapping, other stress-responsive kinases were checked. The signal transduction of the SEK1-stress-activated protein kinase (SAPK) axis was specifically activated in 6-month-old Tg2576 OBs (Figures 7A,B), while no appreciable changes were detected in the activation status of PKA (Figure 7C) and other survival kinases such as FAK, and PDK1/PKC axis with respect to WT animals (Supplementary Figure S2).

**Figure 8:**
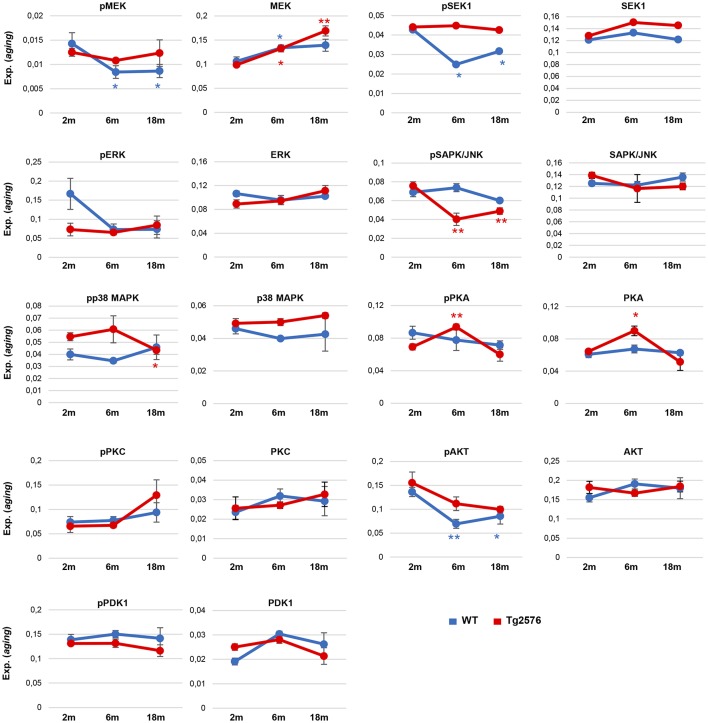
Monitorization of survival kinases during the aging process in WT and Tg2576 OBs. Western-blotting were performed for the kinase panel (total and phosphorylated levels) in the OB from WT and Tg2576 mice of 2, 6, and 18 months of age. Quantitation data were referred to the observed levels in 2-month-old mice for each condition. *P < 0.05 vs. 2-month-old mice; **P < 0.01 vs. 2-month-old mice.

To deepen our understanding of the APP-dependent regulatory effects on kinase dynamics during the aging process in the OB of Tg2576 mice, steady-state levels and phosphorylated isoforms were independently evaluated in WT and Tg2576 mice during aging. For that, protein profiles were quantified in a time-dependent manner at 2-, 6-, and 18-months old (Figure 8). With respect to data obtained at 2 months of age, the activation of downstream ERK, and PDK1/PKC axis were constant in Tg2576 OBs, whereas a drop in the activation status of OB p38 MAPK was observed in 18-month-old Tg2576 mice when AD pathology is well established. No changes were observed in the activation state of olfactory SEK1 during the aging process in Tg2576 mice, in contrast with the inactivation observed in 6-, and 18-month-old WT animals (Figure 8). Conversely, a progressive inactivation was detected in its kinase downstream cascade as evidenced by SAPK dephosphorylation in Tg2576 mice (6-, and 18-month old). In addition, a significant variation was also observed in OB PKA levels in 6-month-old Tg2576.

### Olfactory SEK1/MKK4 and PKA Signaling Are De-regulated Across Human AD Grading

**Figure 9:**
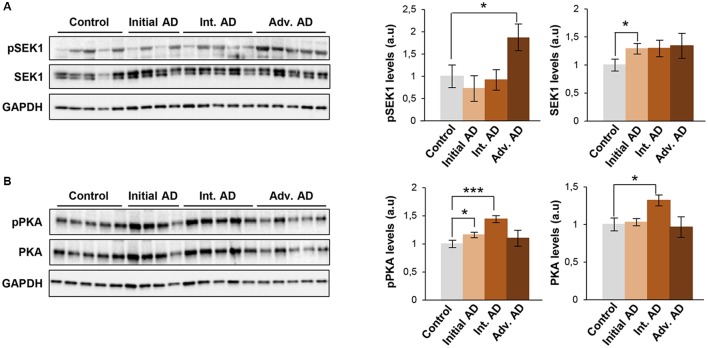
Olfactory SEK1/MKK4 and PKA are differentially activated across Braak stages in human AD. Levels and residue-specific phosphorylation of SEK1/MKK4 **(A)**, and PKA **(B)** in the OB across AD phenotypes. Equal loading of the gels was assessed by Ponceau staining and hybridization with a GAPDH specific antibody. Right panels show histograms of band densities. Data are presented as mean ± SEM from five independent OB samples per group. *P < 0.05 vs. control group; ***P < 0.001 vs. control group. Representative Western blot gels are shown.

Much effort has been spent on studying the role of Aβ in sporadic Alzheimer’s disease (sAD) pathogenesis but the available information is insufficient to fully understand the disease progression at the level of olfactory signaling (Lachén-Montes et al., 2017). To investigate whether SEK1 and PKA signaling pathways perturbed in the OB of Tg2576 mice were also associated with human sAD, the activation state of the corresponding survival pathways was measured by Western blotting in OBs from sAD subjects with different neuropathological grading (Table 1). First, we performed immunohistochemical analysis to localize SEK1 and PKA in human OB. As shown in Supplementary Figures S4A,B, positive staining for the activated form of SEK1 was observed in the OB astrocytes and neurons. However, a specific mild staining for non-phosphorylated SEK1 was observed in neurons (Supplementary Figures S4C,D). With respect to an activated form of PKA, a diffuse staining of neuropil and all OB cellular components was observed (Supplementary Figures S4E,F), even in glial cells from granular layer (Supplementary Figure S4F), while a mild staining in neuropil was observed for the non-phosphorylated PKA (Supplementary Figures S4G,H). As shown in Figure 9, the activation of SEK1 was specifically increased in advanced AD stages (Braak V–VI; Figure 9A). However, PKA activity was significantly increased in the initial (Braak I–II) AD stage, with respect to subjects with normal neuropathological examination (Figure 9B). In intermediate AD stage (Braak III–IV), a significant increment was also observed in total and activated PKA levels (Figure 9B).

## Discussion

We consider that a better understanding of the molecular events affected by the progressive accumulation of amyloid pathology might offer new olfactory targets for earlier diagnosis and therapeutic intervention. In particular, we report that: (i) multiple OB proteotranscriptomic variations appear at pre-plaque stages in Tg2576 mice; (ii) the functional interactome of the hAPPSw isoform at olfactory level is progressively modulated in these mice; (iii) the mitochondrial PHB complex was compromised at early stages in Tg2576 OBs; (iv) specific olfactory signaling routes (Akt, p38 MAPK, SEK1, and SAPK) were modulated in a time-dependent manner respect to WT animals; (v) APP overproduction induced specific regulatory effects on kinase dynamics (SEK1/SAPK, PKA) during the aging process in Tg2576 mice; and (vi) the SEK1 and PKA pathways were differentially activated during human AD grading at the level of the OB. All these findings at the very early pre-plaque stage provided mechanistic clues on the olfactory mechanisms involved in the progression of cognitive deficits previously reported in these mice (Cuadrado-Tejedor and García-Osta, 2014).

To systematically assess the global effect of olfactory APP production on gene expression at the transcript and protein level, we used a combinatorial omics analysis. The minimal overlap observed in transcriptome and proteome remodelings between 2-, and 6-month-old Tg2576 mice support the hypothesis that distinctive pathophysiological processes are involved in the OB during the initial progression of AD-like amyloid pathology. For example, functional clustering suggested that changes in the growth of neurites (in 2-month-old transgenic mice), and microtubule dynamics in the OB (in 6-month-old Tg2576 mice) occur in the absence of neuropathological amyloid plaques, supporting the notion that cytoskeletal remodeling is an early AD pathological hallmark (Do Carmo et al., 2018). Despite the experimental and technical noise in both mRNA and protein measurements, which may contribute to an underestimation of true correlations, RNA-protein correlation was missing in our study. Buffering of mRNA variation against protein levels can occur at multiple levels, including intra- and inter-individual genomic variation (Battle et al., 2015; Liu et al., 2016). The discrepancy we observed may be due to: (i) the use of different set of animals for each technological platform; (ii) the spatial and temporal delayed synthesis between mRNA and protein (Liu et al., 2016); (iii) post-transcriptional events; and (iv) the different hydrophobicity and solubility of specific proteome subsets (e.g., olfactory receptors), which hampers their characterization and quantitation by mass-spectrometry.

Prohibitin deficiencies have been previously associated with neurodegenerative phenotypes (Merkwirth et al., 2012; Dutta et al., 2018). During aging, our data indicated that Phb levels are stable while mutated APP is overproduced, probably to counteract the disease-aggravating oxidative stress during AD progression in Tg2576 mice. Generally, repression/induction of Phb1 is paralleled by a concomitant decrease/increase of its assembly partner Phb2 (Sánchez-Quiles et al., 2010; Merkwirth et al., 2012). Accordingly, OB Phb subunits are functionally interdependent in Tg2576 mice. In contrast, Phb subunits are not interdependent in the OB during AD neurodegeneration in humans (Lachén-Montes et al., 2017), indicating that the tangled regulatory mechanisms that govern the mitochondrial homeostasis in olfactory cells significantly differ between transgenic mice and sporadic human AD. Aberrant regulation of a subset of kinases may represent the triggering events leading to the spread of a perturbed signaling in AD (Perluigi et al., 2016). p38 MAPK is a multifunctional kinase that is activated by Aβ in cultured neurons (Criscuolo et al., 2015), phosphorylates Tau protein (Li et al., 2003; Ferrer et al., 2005), and mediates the Aβ-induced inflammatory activation (Bachstetter et al., 2011). Different alterations of p38 MAPK pathway have been observed in the OB, hippocampus, and cortical areas at early stages in human AD (Hensley et al., 1999; Sun et al., 2003; Munoz and Ammit, 2010; Criscuolo et al., 2015). In Tg2576 mice, we observed an early activation of p38 MAPK (validated by paralleled increase in ATF2 phosphorylation levels), suggesting detrimental effects, such as neuroinflammation and excitotoxicity at the level of the OB (Yi et al., 2018). Although Akt has been recently proposed as a therapeutic target for AD-associated memory impairments (Griffin et al., 2005), different results have been obtained about the Akt activation across brain structures of human AD (Rickle et al., 2004; Petersen et al., 2007; Lachén-Montes et al., 2016). The specific activation of olfactory Akt observed in 6-month-old Tg2576 mice indicates potential protective mechanisms against memory impairments and synaptic deficits (Griffin et al., 2005). In an effort to delineate the oxidative stress signaling events in the OB of Tg2576 mice, we observed an increment in the activation of SAPK/JNK pathway in 6-month-old Tg2576 OBs. This activation precedes the Aβ deposition, although Aβ may enhance its activation at a later time (Zhu et al., 2001). In the human brain, phospho-SAPK is significantly increased in AD over control cases, overlapping with Tau-positive neurofibrillary pathology (Bachstetter et al., 2011; Kelly, 2018). In this study, we have detected an increment in the expression of phosphorylated SEK1 (an upstream activator of the SAPK/JNK route) exclusively in subjects with advanced AD stage (Braak V–VI). Previous reports suggest that this activation may play a role in the tau phosphorylation, and consequently, the formation of NFTs in late AD stages (Zhu et al., 2001). PKA is a tau-kinase and its expression/activity tends to be reduced in different contexts of AD pathology (Liang et al., 2007). In humans, a decrease in PKA activity was observed in the temporal cortex from AD subjects with a Braak stage V–VI (Iulita et al., 2014). However, we report a PKA overactivation that occurs in the OB derived from AD subjects with initial stages (Braak I–II), indicating that cAMP signaling appears to be stage and brain region specific.

Although our study has uncovered many intricacies in OB molecular homeostasis during early stages of AD-related amyloidogenic pathology, there are potential limitations of our study that warrant discussion. First, due to technological issues, we failed to accurately quantify many proteins expressed at low levels that might also participate in the olfactory AD progression in Tg2576 mice. Second, our results are limited by transcript/protein abundance averaging among the multiple cell layers present in the OB, hampering the exploration of olfactory cell-type specific molecular alterations. Third, Aβ, APP, and its derived species may co-exist inside neurons (Crowe et al., 2018), and based on our experimental workflow, we cannot pinpoint which APP-derived species are responsible for the observed molecular disturbances. Finally, this study should be complemented with behavior testing to characterize which molecular abnormalities are directly linked to the cognitive deficit, as well as additional omic studies performed in different brain areas to verify the specificity of the molecular alterations detected at the level of the OB.

## Conclusion

Our dual-omic approach revealed the disruption of multiple molecular pathways at early stages of the OB amyloid pathology, leading to the identification of differential olfactory targets linked to APP metabolism. These findings point out the potential utility of alternative olfactory pathways for disease modification, in a stage-dependent manner, through intranasal therapies (Cheng et al., 2017) based on enzyme replacement or specific drug delivery (Agrawal et al., 2018).

## Ethics Statement

### Human Samples

According to the Spanish Law 14/2007 of Biomedical Research, inform written consent forms of Brain Bank of IDIBELL, and Neurological Tissue Bank of IDIBAPS-Hospital Clinic (Barcelona, Spain) was obtained for research purposes from relatives of patients included in this study. The study was conducted in accordance with the Declaration of Helsinki and all assessments, post-mortem evaluations, and procedures were previously approved by the Clinical Ethics Committee of Navarra Health Service.

### Animals

All animal care and experimental procedures were in accordance with European and Spanish regulations (86/609/CEE; RD1201/2005) and were approved by the Ethical Committee of the University of Navarra (no. 018/05).

## Author Contributions

JF-I and ES designed and supervised the complete study. ML-M, AG-M, MP and KA performed sample preparation, transcriptomic experiments, and functional assays. JF-I performed liquid chromatography-tandem mass spectrometry analysis. MG-O, MZ, IF and AP-M performed immunohistochemical analysis and neuropathological characterizations. ES performed bioinformatics, network biology analysis, data interpretation and wrote the article.

## Conflict of Interest Statement

The authors declare that the research was conducted in the absence of any commercial or financial relationships that could be construed as a potential conflict of interest.

## Footnotes

^1^http://proteomecentral.proteomexchange.org

^2^www.qiagen.com/ingenuity

## Supplementary Material

FIGURE S1 Olfactory β-Amyloid pathology increases with the disease progression in TG2576 mice. OBs were harvested from 2 (**A–C**), 6 (**D–F**) and 14 (**G–J**) month-old Tg2576 mice. Intraneuronal Aβ immunoreactivity can be observed in 2 month-old mice (arrow heads; panel **B** and more detailed in panel **C**). OB samples from 6 month-old animals (panels **D,E** and **F**) shows moderate Aβ deposition in form of diffuse plaques (asterisk). By contrast, mature plaques (asterisk in panel G, H and insert J) and vascular Aβ (insert I) is evident in 14 month-old Tg2576. Scale bars 500 μm for panels **A, D, G**, 100 μm for panels **B, E, H** or 10 μm **(C, F, I, J)**. (GL: glomerular layer; EPL: external plexiform layer; MC: mitral layer; GrL: granular layer; AON: anterior olfactory nucleus).

FIGURE S2 OB PP5 up-regulation in 6-month-old Tg2576 mice (***P* < 0.01 respect to control group) **(A)**. No significant changes were found in FAK and PDK1/PKC signaling axis **(B)**.

FIGURE S3 Visualization of predictive interactions between APP and survival kinases (orange circles) based on differential datasets derived from 2-month-old Tg2576 mice.

FIGURE S4 Immunohistochemical localization of OB SEK1 and PKA (phosphorylated and non-phosphorylated forms). Representative immunohistochemical staining pattern of pSEK1 **(A,B)**, SEK1 **(C,D)**, pPKA **(E,F)** and PKA **(G,H)** in control and AD cases.

TABLE S1 Differential OB transcriptome in 2- and 6-month-old Tg2576 mice respect to WT mice.

TABLE S2 Diffefrential OB proteome in 2- and 6-month-old Th2576 mice respect to WT mice.

TABLE S3 Functional analysis of omics datasets.

## Abbreviations

AD: Alzheimer’s disease
Akt: Protein kinase B
APP: Amyloid precursor protein
ATF2: Activating Transcription Factor 2
CaMKII: Ca^2+^/calmodulin-dependent protein kinase II
ERK: Extracellular signal-regulated kinase
FAK: Focal adhesion kinase
MEK: ERK Activator Kinase
OB: Olfactory bulb
p38 MAPK: p38 mitogen-activated protein kinase
PDK1: Phosphoinositide-dependent protein kinase 1
Phb: Prohibitin
PKA: protein kinase A
PKC: protein kinase C
PP5: Serine/threonine-protein phosphatase 5
SAPK/JNK: stress-activated protein kinase/Jun-amino terminal kinase
SEK1/MKK4: Mitogen-activated protein kinase Kinase 4.
