# Supplementary material for: Early-Onset Molecular Derangements in the Olfactory Bulb of Tg2576 Mice: Novel Insights Into the Stress-Responsive Olfactory Kinase Dynamics in Alzheimer’s Disease
Source: Front Aging Neurosci. 2019 Jun 11;11:141. doi: 10.3389/fnagi.2019.00141 (PMC6579864; doi:10.3389/fnagi.2019.00141)
Supplement: FIGURE S1 — Olfactory β-Amyloid pathology increases with the disease progression in TG2576 mice. OBs were harvested from 2 (A–C), 6 (D–F) and 14 (G–J) month-old Tg2576 mice. Intraneuronal Aβ immunoreactivity can be observed in 2 month-old mice (arrow heads; panel B and more detailed in panel C). OB samples from 6 month-old animals (panels D,E and F) shows moderate Aβ deposition in form of diffuse plaques (asterisk). By contrast, mature plaques (asterisk in panel G, H and insert J) and vascular Aβ (insert I) is evident in 14 month-old Tg2576. Scale bars 500 μm for panels A, D, G, 100 μm for panels B, E, H or 10 μm (C, F, I, J). (GL: glomerular layer; EPL: external plexiform layer; MC: mitral layer; GrL: granular layer; AON: anterior olfactory nucleus). [file Presentation_1.PPTX]

## Slide 1
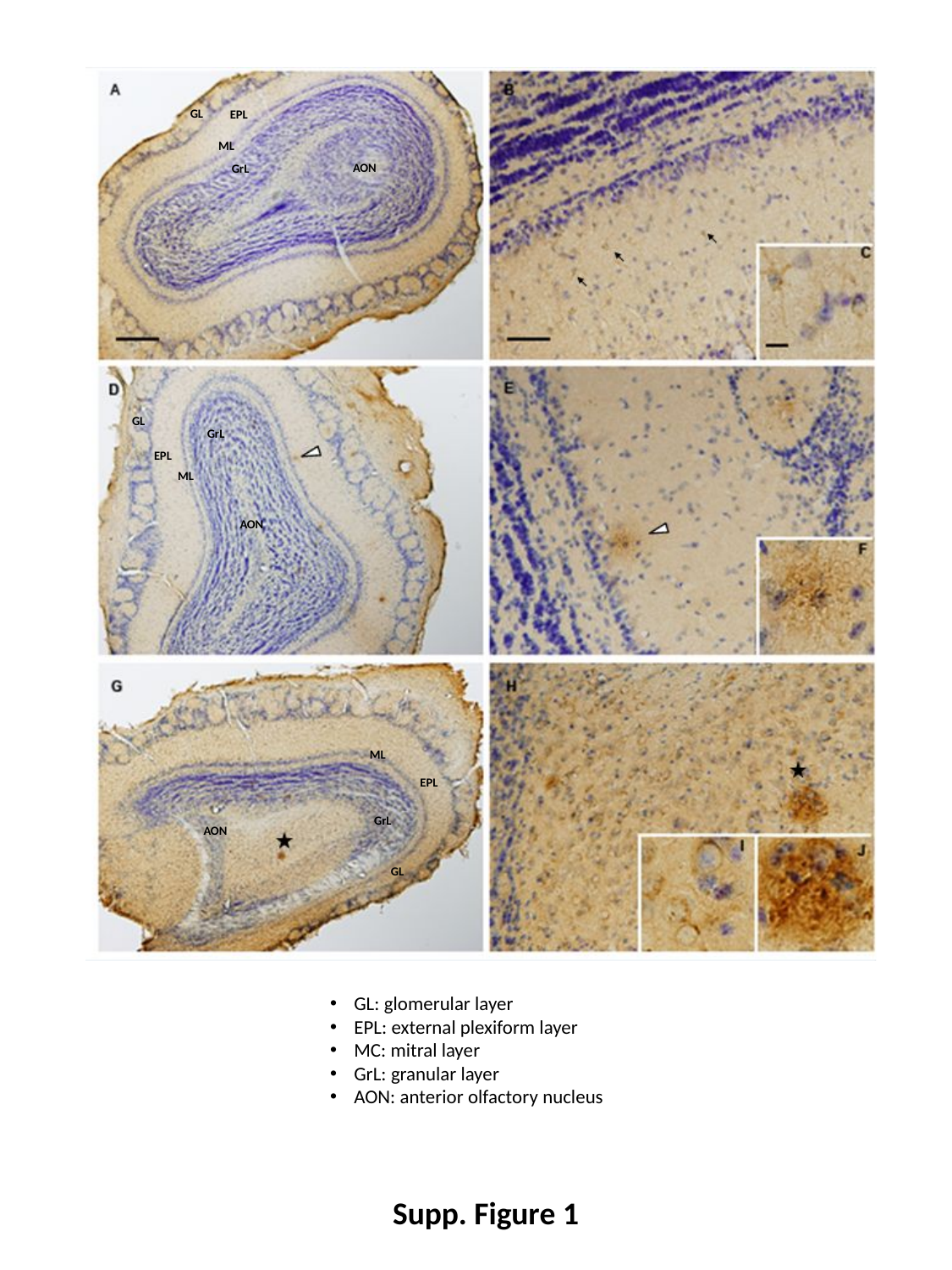

GL
EPL
ML
AON
GrL
GL
GrL
EPL
ML
AON
ML
EPL
GrL
AON
GL
GL: glomerular layer
EPL: external plexiform layer
MC: mitral layer
GrL: granular layer
AON: anterior olfactory nucleus
Supp. Figure 1

## Slide 2
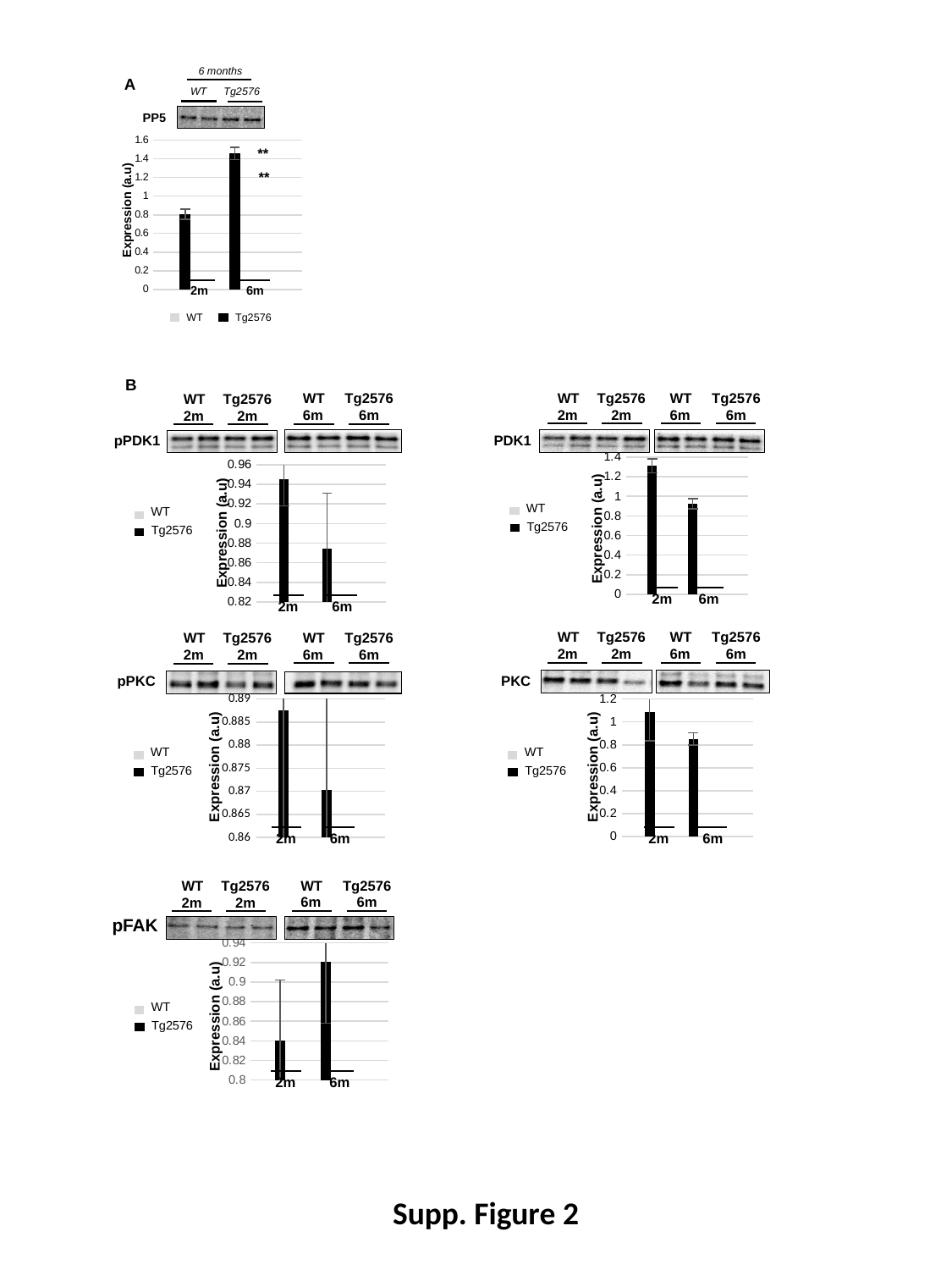

6 months
A
WT
Tg2576
PP5
### Chart
| Category | WT | Tg2576 |
|---|---|---|
| 2 meses | 1.0 | 0.805497825187119 |
| 6 meses | 1.0 | 1.458250043253754 |**
**
Expression (a.u)
2m
6m
WT
Tg2576
B
WT
6m
Tg2576
6m
WT
2m
Tg2576
2m
WT
6m
Tg2576
6m
WT
2m
Tg2576
2m
pPDK1
PDK1
### Chart
| Category | WT | Tg2576 |
|---|---|---|
| 2 meses | 1.0 | 1.309958096986272 |
| 6 meses | 1.0 | 0.923510350661002 |
### Chart
| Category | WT | Tg2576 |
|---|---|---|
| 2 meses | 1.0 | 0.94513131994899 |
| 6 meses | 1.0 | 0.874660503260553 |WT
WT
Tg2576
Expression (a.u)
Tg2576
Expression (a.u)
2m
6m
2m
6m
WT
6m
Tg2576
6m
WT
2m
Tg2576
2m
WT
6m
Tg2576
6m
WT
2m
Tg2576
2m
pPKC
PKC
### Chart
| Category | WT | Tg2576 |
|---|---|---|
| 2 meses | 1.0 | 1.085476936689718 |
| 6 meses | 1.0 | 0.851729943696638 |
### Chart
| Category | WT | Tg2576 |
|---|---|---|
| 2 meses | 1.0 | 0.887579723378775 |
| 6 meses | 1.0 | 0.870366224500105 |WT
WT
Expression (a.u)
Expression (a.u)
Tg2576
Tg2576
2m
6m
2m
6m
WT
6m
Tg2576
6m
WT
2m
Tg2576
2m
pFAK
### Chart
| Category | WT | Tg2576 |
|---|---|---|
| 2 meses | 1.0 | 0.840444559345196 |
| 6 meses | 1.0 | 0.920461432781501 |WT
Expression (a.u)
Tg2576
2m
6m
Supp. Figure 2

## Slide 3
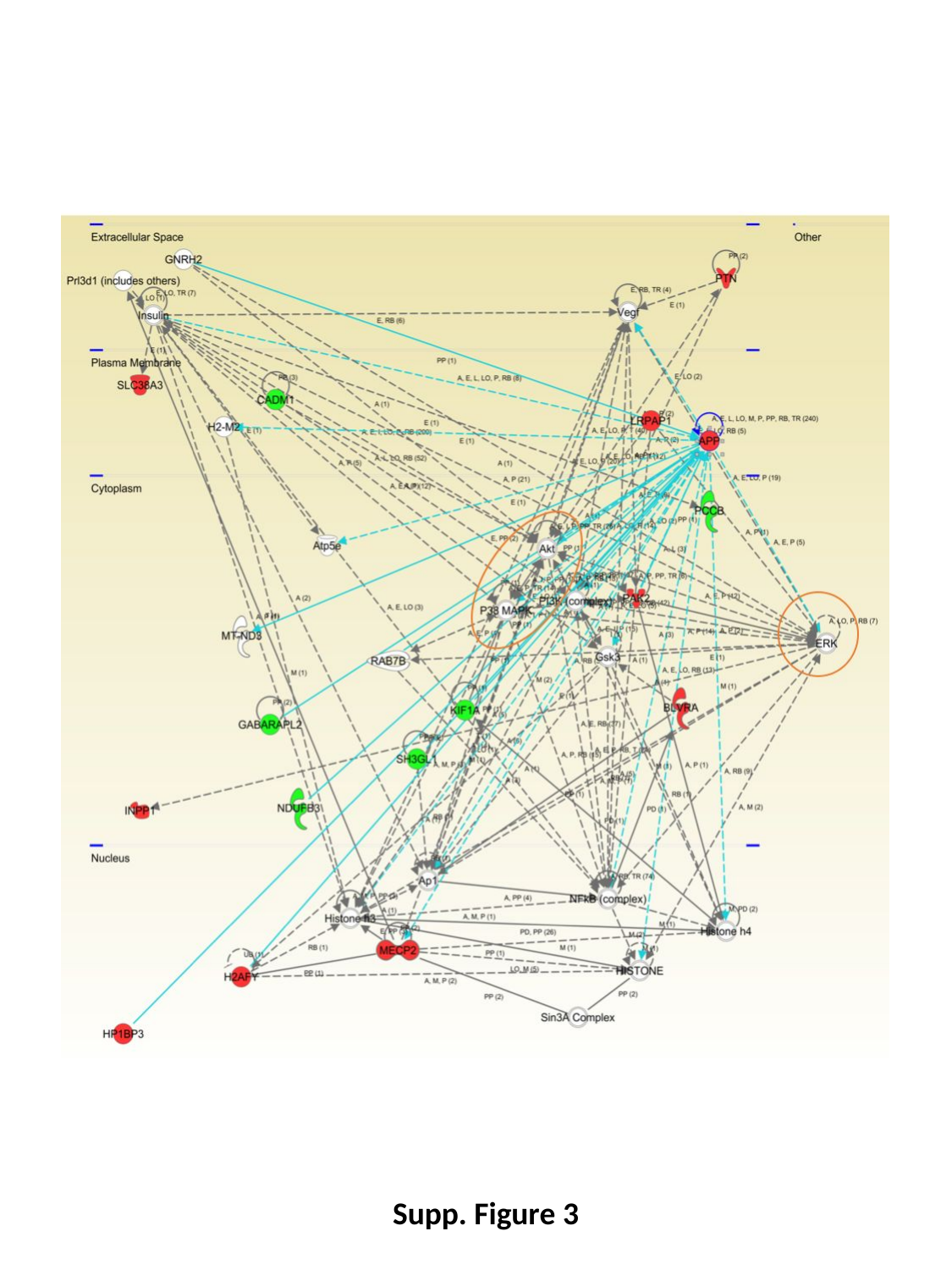

Supp. Figure 3

## Slide 4
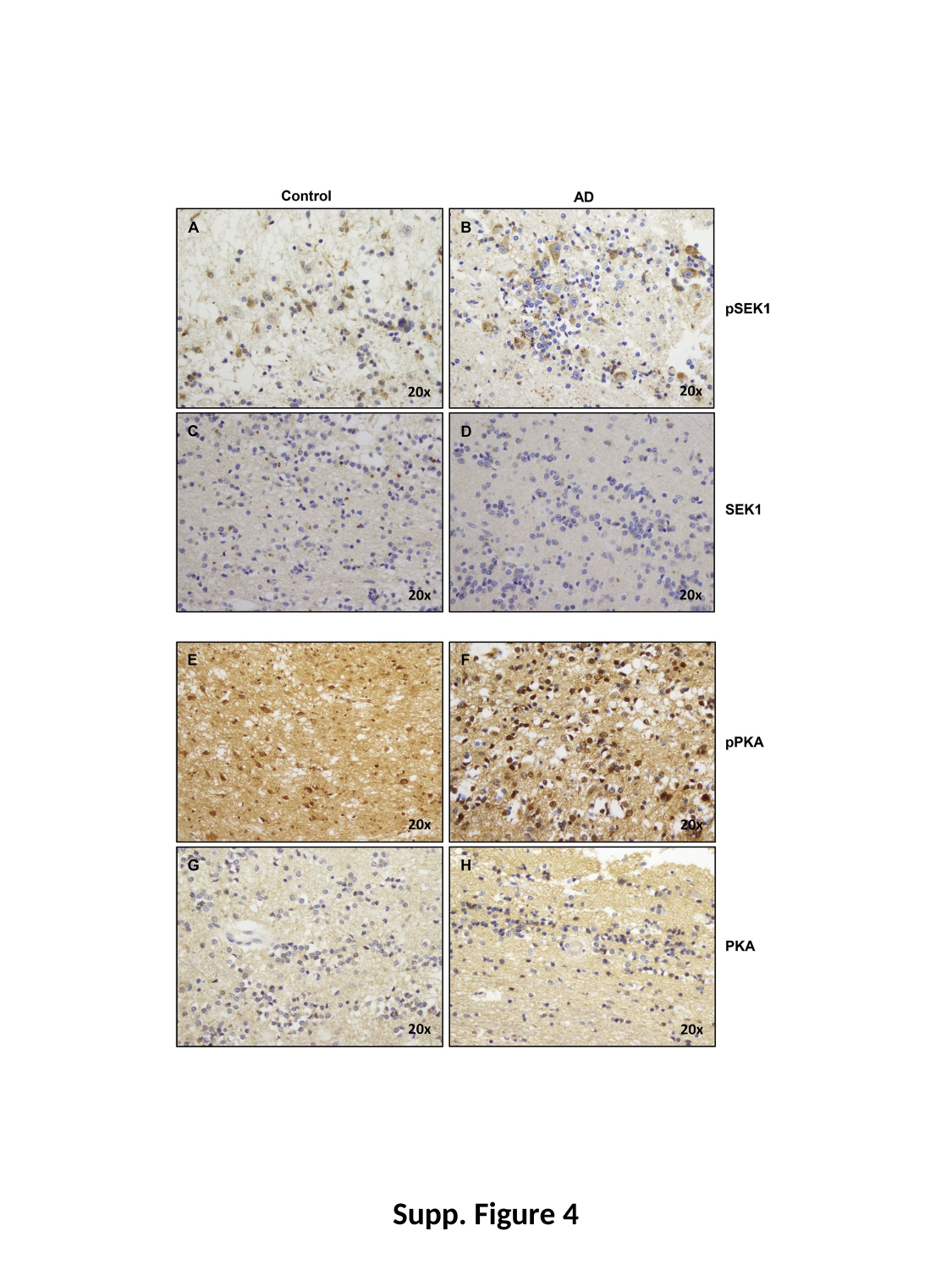

Supp. Figure 4
